# Isolation and Characterization of a *Weizmannia coagulans* Bacteriophage Youna2 and Its Endolysin PlyYouna2

**DOI:** 10.4014/jmb.2303.03021

**Published:** 2023-05-12

**Authors:** Bokyung Son, Youna Kim, Booyoung Yu, Minsuk Kong

**Affiliations:** 1 Department of Food Science and Technology, Seoul National University of Science and Technology, Seoul 01811, Republic of Korea; 2 Department of Food Biotechnology, Dong-A University, Busan 49315, Republic of Korea

**Keywords:** *Weizmannia coagulans*, bacteriophage, endolysin, food spoilage

## Abstract

*Weizmannia coagulans* (formerly *Bacillus coagulans*) is Gram-positive, and spore-forming bacteria causing food spoilage, especially in acidic canned food products. To control *W. coagulans*, we isolated a bacteriophage Youna2 from a sewage sludge sample. Morphological analysis revealed that phage Youna2 belongs to the *Siphoviridae* family with a non-contractile and flexible tail. Youna2 has 52,903 bp double-stranded DNA containing 61 open reading frames. There are no lysogeny-related genes, suggesting that Youna2 is a virulent phage. *plyYouna2*, a putative endolysin gene was identified in the genome of Youna2 and predicted to be composed of a *N*-acetylmuramoyl-L-alanine amidase domain (PF01520) at the N-terminus and unknown function DUF5776 domain (PF19087) at the C-terminus. While phage Youna2 has a narrow host range, infecting only certain strains of *W. coagulans*, PlyYouna2 exhibited a broad antimicrobial spectrum beyond the *Bacillus* genus. Interestingly, PlyYouna2 can lyse Gram-negative bacteria such as *Escherichia coli*, *Yersinia enterocolitica*, *Pseudomonas putida* and *Cronobacter sakazakii* without other additives to destabilize bacterial outer membrane. To the best of our knowledge, Youna2 is the first *W. coagulans*-infecting phage and we speculate its endolysin PlyYouna2 can provide the basis for the development of a novel biocontrol agent against various foodborne pathogens.

## Introduction

Bacteriophages (phages) are bacteria-infecting viruses and known as the most abundant biological entities on Earth [1]. Phages have been considered as an alternative antibacterial agent by their distinguishable features such as high specificity to the target bacteria and harmlessness to humans [2, 3]. In food industry, phages can be an effective and inexpensive tool to fight against foodborne pathogens [4]. The application of phages to various foods has been reported to be successful in preventing foodborne pathogen contamination in recent years and an increasing number of phage products have been commercialized for controlling important foodborne pathogens [4, 5]. Beside the phage itself, endolysins have been also focused as an alternative strategy to control pathogenic bacteria. Endolysins are phage-encoded enzymes that hydrolyze the peptidoglycan of host bacteria at the end of the phage replication cycle, liberating of progeny phage particles from within [6, 7]. Endolysin can be externally applied to lyse bacterial cells, especially Gram-positive bacteria due to the absence of an outer membrane in the cell wall [8]. Consequently, endolysin can access the peptidoglycan and kill unwanted bacteria. In addition, endolysins present several important advantages as biocontrol agents: low chance of developing bacterial resistance, high specificity for target bacteria without disturbing other bacteria, and strong enzymatic activity to rapidly lyse bacterial cells [9, 10]. These features make endolysin a promising and potent antimicrobial candidate, particularly in the light of increasing bacterial drug resistance.

*Bacillus* species are Gram-positive and spore-forming bacteria and one of the bacterial genera with the highest prevalence in the food industry. Despite their numerous advantages in industry applications, they could also be problematic [11]. In particular, *Weizmannia coagulans* (re-classified from *Bacillus coagulans*, [12]) is known to be one of the important food spoilage microorganisms, frequently found in the canned fruit and vegetable processing industry [11, 13]. *W. coagulans* causes flat sour spoilage, drastically acidifying food products, eventually leading to significant economic losses in the food industry [14, 15]. Previously, several methods for controlling *W. coagulans* have been reported including heat treatment in the range from 95-103°C and thermochemical treatment using essential oils [16]. Also, butyric acid vanillyl ester or caprylic acid vanillyl ester showed antibacterial activity against *W. coagulans* [17]. However, these control methods could negatively affect the quality and sensory properties of foods, necessitating alternative antibacterial tools that inhibit the growth of these bacteria. Moreover, as the rapid emergence of antibiotic-resistant bacteria has become a global concern, it becomes important to develop a new type of control agent. To address these issues, here we isolated a novel *W. coagulans*-infecting phage, Youna2 and characterized its endolysin PlyYouna2. The endolysin gene, *plyYouna2* was cloned and overexpressed in *Escherichia coli*, and the purified endolysin was biochemically characterized. PlyYouna2 showed strong lytic activity against *W. coagulans* and a number of other Gram-positive and Gram-negative bacteria, suggesting that PlyYouna2 has a relatively broad antibacterial spectrum. The results demonstrate that PlyYouna2 can be a promising antimicrobial agent against various foodborne pathogens.

## Materials and Methods

### Bacterial Strains and Growth Conditions

Table 1 shows the list of bacterial strains used in this study. *W. coagulans* KACC 15983 was used to isolate and propagate the phage Youna2. All *W. coagulans* strains were grown in tryptic soy broth (TSB, BD Difco, USA) at 50°C, and other Gram-positive bacteria including *Bacillus* and *Staphylococcus* strains were grown in TSB at 37°C. *Clostridium perfringens* cells were grown at 30°C under anaerobic conditions in brain heart infusion medium (BHI, BD Difco). Unless otherwise noted, cells were grown in Luria-Bertani (LB, BD Difco) broth at 37°C.

### Bacteriophage Isolation and Propagation

Bacteriophage Youna2 was isolated from the secondary sludge of Jungnang Water Recycle Center in Seoul, South Korea. The sludge samples were mixed with 2X TSB in a 1:1 ratio and the overnight culture of *Weizmannia coagulans* KACC 15983 was added to the mixture in the presence of 0.5 mM CaCl_2_ and 0.5 mM MgCl_2_, followed by incubation at 50°C at 250 rpm overnight. Then, the culture was harvested, and the supernatant was filtered using a 0.45 uM filter. The presence of phage in the filtrate was confirmed by spotting 10 μl of tenfold serially diluted filtrates onto soft agar (TSB containing 0.4% agar) containing 100 μl of host *W. coagulans* culture and 0.5 mM CaCl_2_ and 0.5 mM MgCl_2_. The plates were incubated overnight at 50°C, and the formation of plaques was monitored. Clear plaques were picked with a sterile tip and eluted in SM buffer (Sodium-Magnesium Sulfate buffer). The plaque isolation steps were repeated at least three times as described above.

For phage propagation, *W. coagulans* strain was incubated at 50°C with shaking at 250 ×*g* until it reached an OD_600_ of 0.7. Subsequently, phages at a multiplicity of infection (MOI) of 0.05 were added with 0.5 mM CaCl_2_ and 0.5 mM MgCl_2_ followed by a 3-h incubation at 50°C. The phage stock solution was prepared with the propagated phages as previously described [18].

### Morphological Analysis by TEM

The morphology of Youna2 was analyzed using Energy-Filtering Transmission Electron Microscope (EF-TEM) as previously described [18]. Youna2 was identified and classified according to the guidelines of the International Committee on Taxonomy of Viruses [19].

### Host Range Analysis

The bacterial strains used for host range analysis were listed in Table 1. Each bacterial culture was mixed with 5 ml of TSB or LB soft agar and overlaid on tryptic soy agar (TSA) or LB agar plates. Subsequently, 10-fold diluted bacteriophage Youna2 lysates (10^10^ to 10^3^ plaque-forming units (PFUs)/ml) were spotted onto the plates and incubated overnight. The infectivity of Youna2 was determined based on the appearance of the plaques: “+”, clear single plaques observed; “-”, no lysis nor growth inhibition.

### Bacteriophage DNA Extraction

Bacteriophage genomic DNA was purified as previously described [20].

### Full-Genome Sequencing

Genomic DNA of phage Youna2 was sequenced using the Illumina MiSeq system at Sanigen Inc., South Korea and the *de novo* assembly algorithm of CLC Genomics Workbench 10.0.1 was used for genome assembly. Open reading frames (ORFs) annotation was conducted using the Rapid Annotation using Subsystem Technology (RAST) pipeline, as described previously [21]. This annotation was further complemented using BLASTP [22] and Interproscan. The genome map was created by CGview server [23] and the complete genome sequence of *W. coagulans* phage Youna2 was deposited in GenBank (Accession number OM293949).

### Prediction of PlyYouna2 Structure by AlphaFold2

The protein structure of PlyYouna2 was predicted by AlphaFold2 version 2.3.0. The parameters used in this study: model_preset=monomer and db_preset=reduced_dbs [24]. Structures were visualized using PyMOL 1.7.1.5 [25].

### Cloning, Expression, and Purification of PlyYouna2

The predicted endolysin gene (*plyYouna2*) was amplified from the genomic DNA of the bacteriophage Youna2 by polymerase chain reaction (PCR) using primers, fBamH_PlyYouna2 (5’-GCG GGA TCC ATG GCA AGT AAA CCA TTG TTG GTG ATT G-3’) and rHind_PlyYouna2 (5’-GCG AAG CTT TTA CCT CCT CTT CAC TGT GAC GTA CTT AGG TG-3’). The PCR product was cloned into pET-28a (Novagen, USA), which has an N-terminal hexahistidine (His)-tag sequence. Plasmid with a correct insert was transformed into competent *E. coli* BL21 RIPL (DE3). Cells were grown in 2X YT broth with 50 μg/ml kanamycin and chloramphenicol to an OD_600_ to 0.6–0.8, and the expression of the recombinant PlyYouna2 was induced with 0.5 mM isopropyl-b-d-thiogalactopyranoside (IPTG), followed by incubation for an additional 24 h at 18°C. Bacterial cells were resuspended in lysis buffer (50 mM Tris-HCl, 500 mM sodium chloride and 30% glycerol, pH 8.0) and disrupted by sonication (Branson Ultrasonics, China). After centrifugation at 21,000 ×*g* for 1 h, the supernatant was obtained and filtered with 0.22 μm filter. The filtered supernatant was mixed with nickel-nitrilotriacetic acid (Ni-NTA) agarose (Qiagen, Germany) and incubated with rocking at 4°C for 1 h. After the flow-through was discarded, the resin was serially washed using lysis buffer containing 10 mM imidazole, followed by lysis buffer with 20 mM imidazole. The protein was eluted using an elution buffer (50 mM Tris-HCl, 500 mM sodium chloride, and 200 mM imidazole, pH 8.0). The buffer of the eluted PlyYouna2 was changed to the lysis buffer using Zeba Spin Desalting Columns (MWCO 7 kDa, Thermo Fisher Scientific, USA) and stored at -80°C until use.

### Lytic Activity Assay

The lytic activity of PlyYouna2 was assessed by the turbidity assay [26]. Briefly, exponentially grown *W. coagulans* KACC 10117 was harvested and resuspended in reaction buffer (20 mM Tris-HCl, pH 8.0). PlyYouna2 (final molar concentrations of 0.4, 0.8, 1.2, and 1.6 μM) were incubated with the prepared bacterial cells, the OD_600_ values were monitored for 1 h at room temperature. To evaluate the susceptibility of the target bacterial cells to PlyYouna2 in various pHs, 364 nM of PlyYouna2 was mixed with *W. coagulans* KACC 10117 cell suspensions in Britton-Robinson universal buffer (0.04 M H3PO_4_, 0.04 M H_3_BO_3_, 0.04 M CH_3_COOH, and 0.2 M NaCl). The thermal stability of the endolysin was tested by the lysis assays after the enzyme itself was incubated for 15 min at different temperatures. The effect of NaCl on lytic activity was evaluated at various NaCl concentrations (0–800 mM) [26, 27]. The relative activities were calculated by dividing the ΔOD_600_ of each condition by the ΔOD_600_ of the condition that showed the maximal activity.

## Results and Discussion

### Isolation and Characterization of *W. coagulans* Phage Youna2

**Fig. 1:**
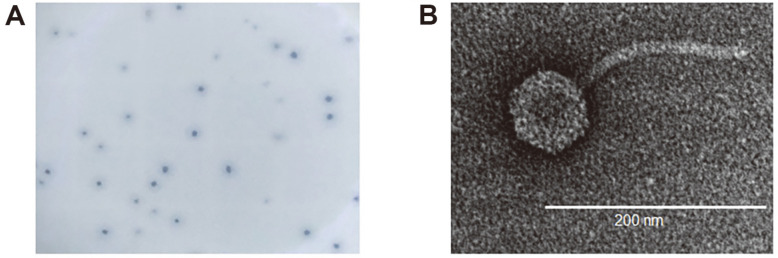
(A) Plaque morphology of Youna2. (B) Transmission electron microscopy image of Youna2. The phage belongs to the family *Siphoviridae*. The scale bar represents 200 nm.

The *W. coagulans* phage Youna2 was newly isolated from sewage sludge samples. This phage formed clear plaques with a halo against *W. coagulans* KACC 15983, a bacterial host strain, implying that tail proteins of Youna2 may have depolymerase activity (Fig. 1A) [28]. EF-TEM revealed that phage Youna2 possesses an icosahedral head with a diameter of 50 nm and a non-contractile and flexible tail with a length of approximately 200 nm, indicating that phage Youna2 belongs to the family *Siphoviridae* (Fig. 1B). We performed a plaque assay to determine the host range of phage Youna2. As shown in Table 1, Youna2 was only able to show infectivity against *W. coagulans* strains, showing plaques on the lawn of the 4 out of 5 W. coagualns strains tested. Other *Bacillus* strains, Gram-positive bacteria or Gram-negative bacteria exhibited resistance to the phage Youna2, indicating that the infectivity of Youna2 is highly specific to its host. This finding explains one of the important characteristics of the phage, host specificity.

### Genome Aanalysis of Phage Youna2

**Fig. 2:**
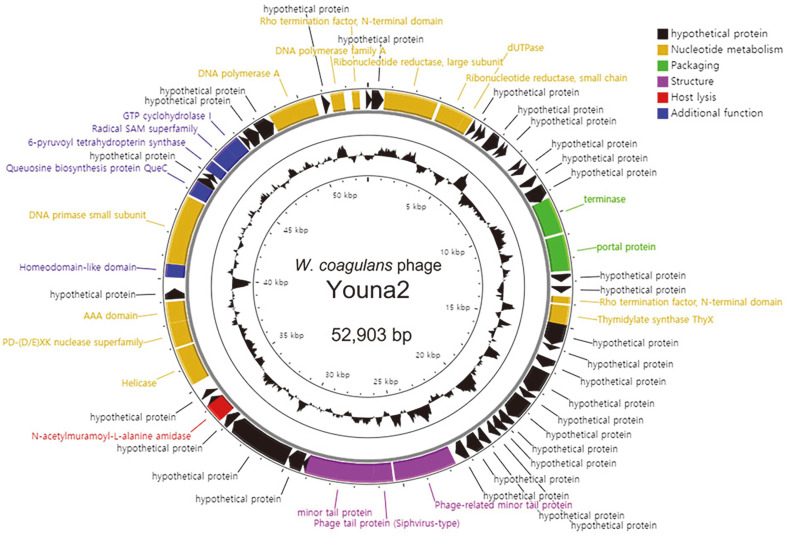
Circular representation of the *W. coagulans* phage Youna2 genome. Predicted ORFs are arranged on the Youna2 genome. Functional groups are categorized into colors as shown in the legend. The inner track represents the GC content plot of the phage.

Whole genome sequencing analysis revealed genomic features of phage Youna2 (Fig. 2). Youna2 is a double-stranded DNA (dsDNA) virus with 52,903-base-pairs. The average G + C content of the genome is 42.72%, and it contains 61 ORFs with no rRNA or tRNA identified. The majority of the predicted genes (38 ORFs) encode hypothetical proteins with unknown functions. We assume that Youna2 is a virulent phage based on the fact that no lysogeny-related genes such as excisionase, genome attachment site (*attP*), repressor, integrase and transposase were identified in the genome of Youna2 [29]. In addition, no genes related to bacterial virulence were identified. BLASTN revealed that the whole nucleotide sequence of Youna2 shares similarity with only four *Bacillus* phages (BSTP12, BSTP10, BSTP8, and BSTP5), with 4% query coverage and 75% identity, suggesting that phage Youna2 is a novel phage. Among the ORFs, phage tail protein which is a *Siphovirus*-type is identified in the genome, supporting that Youna2 belongs to the family *Siphoviridae*. Also, Youna2 encodes a gene involved in the host cell lysis, which is annotated as *N*-acetylmuramoyl-L-alanine amidase.

### Identification and Overexpression of Youna2 Phage Endolysin

**Fig. 3:**
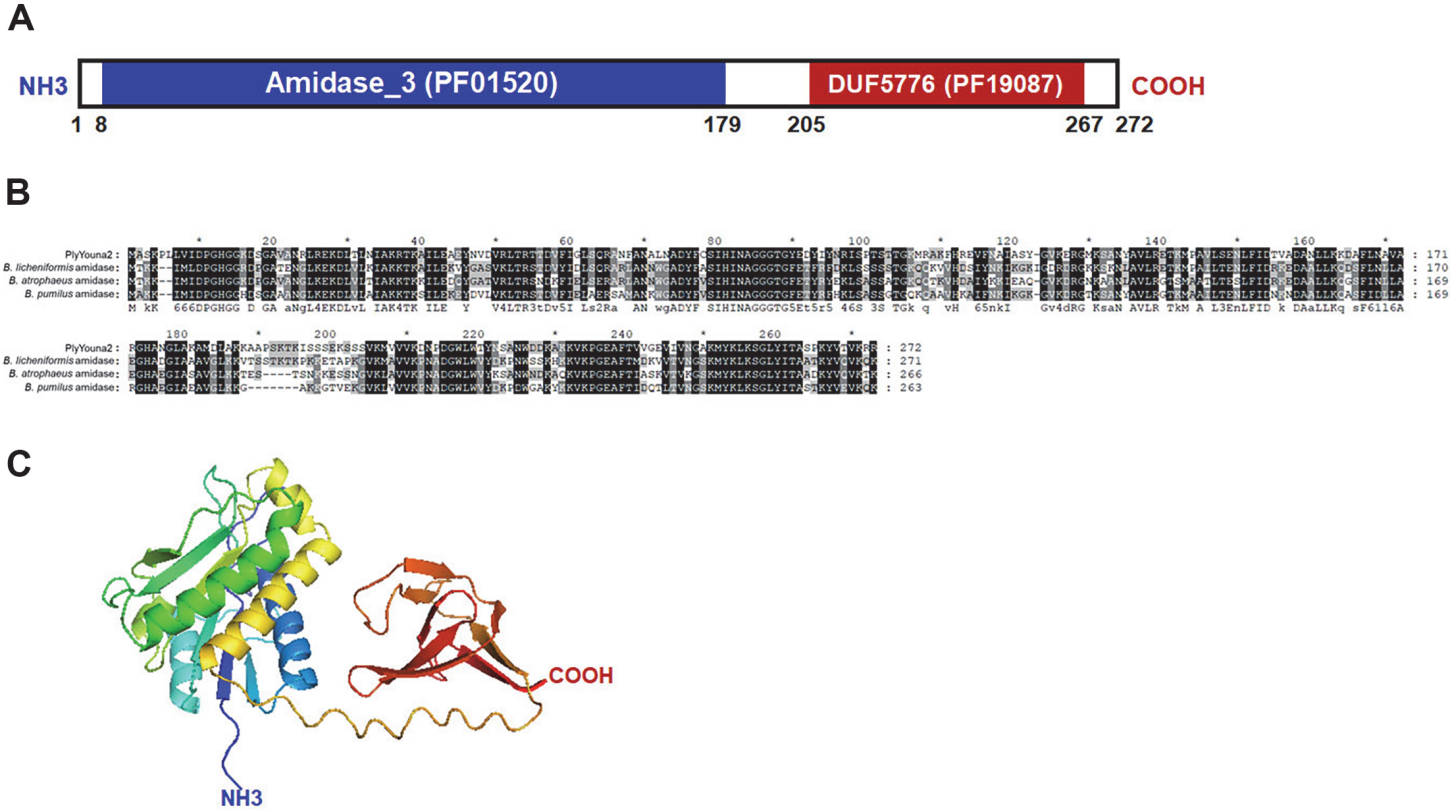
Modular structure of *W. coagulans* phage endolysin PlyYouna2. (**A**) PlyYouna2 domain organization that shows the locations of the predicted N-terminal amidase domain and the predicted C-terminal DUF5776 domain. Numbers indicate residue positions. (**B**) Sequence alignment of PlyYouna2-related enzymes. *N*-acetylmuramoyl-L-alanine amidase from *B. licheniformis*, *B. atrophaeus*, and *B. pumilus*. Conserved and identical residues are shaded in gray (dark gray, >70% conserved; light gray, >40% conserved) and black, respectively. (**C**) Cartoon representation of the PlyYouna2 3D structure predicted by AlphaFold2, colored from the N-terminus (blue) to the C-terminus (red). The N-terminal Amidase_3 and Cterminal DUF5776 domains are indicated.

**Fig. 4:**
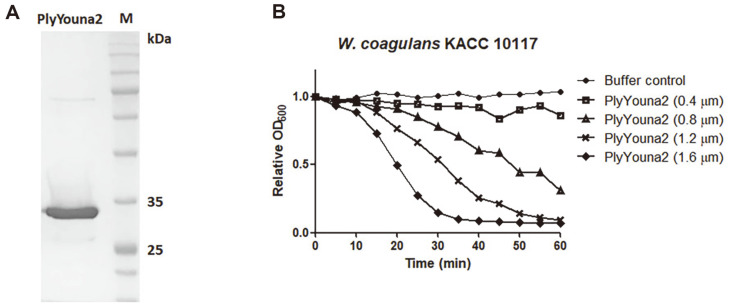
(A) Purified PlyYouna2 was loaded on SDS-PAGE gels. Lane M, protein size marker. (B) Lysis of *W. coagulans* KACC 10117 with four different concentrations of PlyYouna2. The graph is representative of three independent replicates.

As described above, an 819 bp-long endolysin gene was identified from the whole genome sequence of phage Youna2 and is designated as *plyYouna2*. Functional domain analysis using InterProScan database predicted that PlyYouna2 is composed of the two domains with an N-terminal *N*-acetylmuramoyl-L-alanine amidase (8-179 aa) and C-terminal domain of unknown function DUF5776 (205-267 aa) (Fig. 3A). BLASTP analysis showed that the amino acid sequence is highly homologous to several *N*-acetylmuramoyl-L-alanine amidases (E.C. 3.5.1.28), sharing ~60% identity but most of them are from *Bacillus* strains such as *B. licheniformis*, *B. atrophaeus*, and *B. pumilus* (Fig. 3B). Considering that these bacterial autolysins have previously shown to cut the amide bond that connects the peptide and sugar moieties of the peptidoglycan [12], we assume that PlyYouna2 may have the same enzymatic activity. We obtained the predicted PlyYouna2 3D structure using AlphaFold2, an advanced tool for protein structure prediction with high confidence [24]. AlphaFold analysis showed that PlyYouna2 has two distinct domains with a linker in between (Fig. 3C). The C-terminus domain of PlyYouna2 forms six β-sheets and the N-terminus domain comprised five α-helices and six β-sheets. The *plyYouna2* gene was overexpressed in *E. coli* with an N-terminal His6-tag. The purified PlyYouna2 was confirmed by sodium dodecyl sulfate polyacrylamide gel electrophoresis (SDS-PAGE) (Fig. 4A). The turbidity assay showed the strong lytic activity of PlyYouna2 against *W. coagulans* KACC10117 in a concentration-dependent manner (Fig. 4B). When 1.6 μM Youna2 was incubated with the cell suspension, the turbidity (OD_600_) dropped to the basal level of detection within 30 min. Consequently, the results demonstrate that PlyYouna2 is a phage endolysin that can lyse *W. coagulans* cells and could be successfully applied exogenously as an effective antibacterial agent.

### Antibacterial Spectrum of PlyYouna2

The antimicrobial spectrum of PlyYouna2 was tested against 5 *Weizmannia* strains, 9 *Bacillus* strains and other Gram-positive and Gram-negative bacteria (Table 1). All tested *Weizmannia* strains and *Bacillus* strains, except for B. cereus were lysed by PlyYouna2. These results are consistent with the idea that endolysin has a broader antibacterial spectrum than phage since endolysin targets the peptidoglycan of the bacterial cell wall [9]. *Geobacillus stearothermophilus*, *Listeria innocua*, and *L. monocytogenes* were also susceptible to PlyYouna2. Interestingly, PlyYouna2-mediated cell lysis against Gram-negative bacteria including *E. coli*, *Pseudomonas putida*, *Cronobacter sakazakii*, and *Yersinia enterocolitica* was observed without the addition of outer membrane permeabilizers. Since PlyYouna2 is highly positive in charge (pI = 9.78) in the buffer condition we tested, its positive charge seems to be responsible for its interaction with negatively charged bacterial membrane, followed by destabilizing the membrane and killing bacteria cells [30, 31]. Recently, a number of endolysins that naturally lyse Gram-negative bacteria have been reported despite the fact that endolysins generally cannot access the peptidoglycan of Gram-negative bacteria [32, 33]. In these cases, it has been suggested that the presence of polyhistidine tags or N-terminal transmembrane regions of endolysin could be responsible for the lysis of Gram-negative bacteria but the outer membrane permeation mechanisms of the endolysins are still unclear [32, 34, 35].

### Effect of NaCl, pH, and Temperature on the Lytic Activity of PlyYouna2

**Fig. 5:**
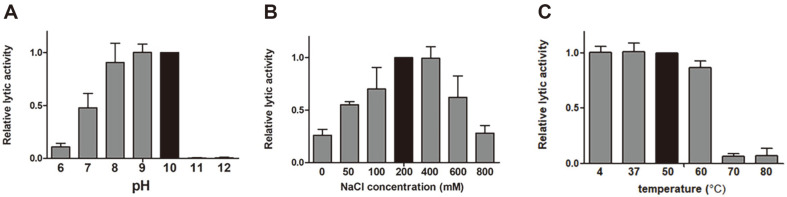
Biochemical characterization of PlyYouna2. The effects of NaCl concentration (**A**), pH (**B**), and temperature (**C**) on the lytic activity of PlyYouna2 against *W. coagulans* KACC 10117 cells are shown. Each column represents the mean of triplicate experiments, and error bars indicate the standard deviation.

The relative lytic activity of PlyYouna2 was evaluated at different concentrations of NaCl, ranging from 0 to 800 mM and the assay was performed against *W. coagulans* KACC 10117 cells for 20 min at room temperature (Fig. 5A). PlyYouna2 exhibited the maximal activity in the presence of 200-400 mM NaCl. At concentrations higher than 400 mM NaCl, PlyYouna2 gradually lost its activity as the concentrations increase. PlyYouna2 was active in the pH range of 7.0–10.0, with the highest activity at pH 10.0 (Fig. 5B). However, no lytic activity was observed at pH 11 or pH 12. The thermostability of PlyYouna2 was determined at various temperatures. While PlyYouna2 retained 80% of its lytic activity after 15 min incubation at 4-60°C, the lytic activity of PlyYouna2 dramatically dropped when it was incubated at 70°C or higher (Fig. 5C).

In conclusion, Youna2 is the first virulent bacteriophage that infects the food spoilage bacterium, *W. coagulans*. An endolysin gene, *plyYouna2* was identified in the genome of Youna2 and characterized. PlyYouna2 showed a broad antimicrobial spectrum and can even lyse Gram-negative bacteria without any outer membrane permeabilizers. These results suggest that PlyYouna2 could be used as a promising biocontrol agent against various foodborne pathogens including Gram-negative as well as Gram-positive bacteria.

## Figures and Tables

**Table 1:** Antimicrobial spectrum of the Youna2 and PlyYouna2.

Bacterial strain			Youna2 Plaque formation^1^	PlyYouna2 Lytic activity^2^
Gram-positive *Weizmannia*	*W. coagulans*	KACC 10117	+	+++
	*W. coagulans*	KACC 11248	-	++
	*W. coagulans*	KACC 15983	+	+
	*W. coagulans*	KACC 18681	+	+
	*W. coagulans*	KACC 15983M	+	+++
Gram-positive *Bacillus*	*B. amyloliquefaciens*	KACC 19163	-	+
	*B. amyloliquefaciens*	KACC 15877	-	++
	*B. cereus*	NCCP 10841 (ATCC 14579)	-	-
	*Bacillus circulans*	JCM 2504	-	+
	*Bacillus licheniformis*	JCM 2505	-	+
	*Bacillus megaterium*	JCM 2506	-	+++
	*Bacillus mycoides*	ATCC 6462	-	+
	*Bacillus pumilus*	JCM 2508	-	+
	*Bacillus subtilis*	ATCC 23857	-	++
	*Bacillus thuringiensis*	ATCC 10792	-	+
Gram-positive *Levilactobacillus*	*Levilactobacillus brevis*	KACC 14481	-	+
	*Levilactobacillus brevis*	KACC 18270	-	++
	*Levilactobacillus brevis*	KACC 16521	-	++
	*Levilactobacillus brevis*	KACC 10553	-	+
	*Levilactobacillus brevis*	KACC 11433	-	++
Other Gram-positive bacteria	*C. perfringens*	NCCP 15911 (FORC 25)	-	-
	*G. stearothermophilus*	ATCC 10149	-	+++
	*Staphylococcus epidermidis*	ATCC 35983	-	-
	*S. aureus*	Newman	-	-
	*Listeria innocua*	ATCC 33090	-	+
	*Listeria monocytogenes*	ATCC 15313	-	+
	*Enterococcus faecalis*	ATCC 10100	-	-
Gram-negative bacteria	*E. coli*	MG 1655	-	++
	*E. coli* O157:H7	ATCC 35150	-	-
	*Cronobacter sakazakii*	ATCC29544	-	+
	*Klebsiella pneumoniae*	KCTC 2242	-	-
	*Pseudomonas aeruginosa*	ATCC 27853	-	+
	*Pseudomonas putida*	KCTC 1643	-	++
	*Salmonella Enteritidis*	ATCC 13076	-	-
	*Salmonella Typhimurium*	UK1	-	-
	*Shigella flexneri*	2a strain 2457T	-	-
	*Yersinia enterocolitica*	ATCC 55075	-	+

^1^Clear plaques, +; no plaque, -

^2^The percentage of lytic activity was obtained by the turbidity reduction assay for 1 h; 1-30% +, 30-70% ++; 71-100% +++, 0% -.

## References

[1] Clokie MR, Millard AD, Letarov AV, Heaphy S (2011). Phages in nature. Bacteriophage.

[2] Garcia P, Martinez B, Obeso J, Rodriguez A (2008). Bacteriophages and their application in food safety. Lett. Appl. Microbiol..

[3] Lu TK, Koeris MS (2011). The next generation of bacteriophage therapy. Curr. Opin. Microbiol..

[4] Połaska M, Sokołowska B (2019). Bacteriophages-a new hope or a huge problem in the food industry. AIMS Microbiol..

[5] Goodridge LD, Bisha B (2011). Phage-based biocontrol strategies to reduce foodborne pathogens in foods. Bacteriophage.

[6] Schmelcher M, Donovan DM, Loessner MJ (2012). Bacteriophage endolysins as novel antimicrobials. Future Microbiol..

[7] Bai J, Kim YT, Ryu S, Lee JH (2016). Biocontrol and rapid detection of food-borne pathogens using bacteriophages and endolysins. Front. Microbiol..

[8] Fischetti VA (2008). Bacteriophage lysins as effective antibacterials. Curr. Opin. Microbiol..

[9] Borysowski J, Weber-Dąbrowska B, Górski A (2006). Bacteriophage endolysins as a novel class of antibacterial agents. Exp. Biol. Med..

[10] Loessner MJ (2005). Bacteriophage endolysins-current state of research and applications. Curr. Opin. Microbiol..

[11] André S, Vallaeys T, Planchon S (2017). Spore-forming bacteria responsible for food spoilage. Res. Microbiol..

[12] Kuroda A, Sugimoto Y, Funahashi T, Sekiguchi J (1992). Genetic structure, isolation and characterization of a *Bacillus licheniformis* cell wall hydrolase. Mol. Gen. Genet..

[13] Lucas R, Grande MJ, Abriouel H, Maqueda M, Omar NB, Valdivia E (2006). Application of the broad-spectrum bacteriocin enterocin AS-48 to inhibit *Bacillus coagulans* in canned fruit and vegetable foods. Food Chem. Toxicol..

[14] Konuray G, Erginkaya Z (2018). Potential use of *Bacillus coagulans* in the food industry. Foods.

[15] Gupta RS, Patel S, Saini N, Chen S (2020). Robust demarcation of 17 distinct *Bacillus* species clades, proposed as novel *Bacillaceae* genera, by phylogenomics and comparative genomic analyses: description of Robertmurraya kyonggiensis sp. nov. and proposal for an emended genus *Bacillus* limiting it only to the members of the Subtilis and Cereus clades of species. Int. J. Syst. Evol. Microbiol..

[16] Haberbeck LU, da Silva Riehl CA, Salomão BdCM, De Aragao GMF (2012). *Bacillus coagulans* spore inactivation through the application of oregano essential oil and heat. LWT-Food Sci. Technol..

[17] Kim JJ, Kim HK (2021). Antioxidant and antibacterial activity of caprylic acid vanillyl ester produced by lipase-mediated transesterification. J. Microbiol. Biotechnol..

[18] Lee JH, Shin H, Son B, Heu S, Ryu S (2013). Characterization and complete genome sequence of a virulent bacteriophage B4 infecting food-borne pathogenic *Bacillus cereus*. Arch. Virol..

[19] Adams MJ, Lefkowitz EJ, King AM, Harrach B, Harrison RL, Knowles NJ (2017). 50 years of the International Committee on Taxonomy of Viruses: progress and prospects. Arch. Virol..

[20] Wilcox S, Toder R, Foster J (1996). Rapid isolation of recombinant lambda phage DNA for use in fluorescence in situ hybridization. Chromosome Res..

[21] McNair K, Aziz RK, Pusch GD, Overbeek R, Dutilh BE, Edwards R (2018). Phage genome annotation using the RAST pipeline. Methods Mol. Biol..

[22] Altschul SF, Madden TL, Schäffer AA, Zhang J, Zhang Z, Miller W (1997). Gapped BLAST and PSI-BLAST: a new generation of protein database search programs. Nucleic Acids Res..

[23] Grant JR, Stothard P (2008). The CGView Server: a comparative genomics tool for circular genomes. Nucleic Acids Res..

[24] Jumper J, Evans R, Pritzel A, Green T, Figurnov M, Ronneberger O (2021). Highly accurate protein structure prediction with AlphaFold. Nature.

[25] DeLano WL (2002). Pymol: an open-source molecular graphics tool. CCP4 Newsl. Protein Crystallogr..

[26] Son B, Yun J, Lim JA, Shin H, Heu S, Ryu S (2012). Characterization of LysB4, an endolysin from the *Bacillus* cereus-infecting bacteriophage B4. BMC Microbiol..

[27] Ha E, Son B, Ryu S (2018). *Clostridium perfringens* virulent bacteriophage CPS2 and its thermostable endolysin lysCPS2. Viruses.

[28] Knecht LE, Veljkovic M, Fieseler L (2020). Diversity and function of phage encoded depolymerases. Front. Microbiol..

[29] Loessner MJ, Inman RB, Lauer P, Calendar R (2000). Complete nucleotide sequence, molecular analysis and genome structure of bacteriophage A118 of Listeria monocytogenes: implications for phage evolution. Mol. Microbiol..

[30] Shavrina M, Zimin A, Molochkov N, Chernyshov S, Machulin A, Mikoulinskaia G (2016). In vitro study of the antibacterial effect of the bacteriophage T5 thermostable endolysin on *Escherichia coli* cells. J. Appl. Microbiol..

[31] Lood R, Winer BY, Pelzek AJ, Diez-Martinez R, Thandar M, Euler CW (2015). Novel phage lysin capable of killing the multidrugresistant gram-negative bacterium *Acinetobacter baumannii* in a mouse bacteremia model. Antimicrob. Agents Chemother..

[32] Lim JA, Shin H, Heu S, Ryu S (2014). Exogenous lytic activity of SPN9CC endolysin against gram-negative bacteria. J. Microbiol. Biotechnol..

[33] Gontijo MTP, Jorge GP, Brocchi M (2021). Current status of endolysin-based treatments against Gram-negative bacteria. Antibiotics.

[34] Antonova NP, Vasina DV, Lendel AM, Usachev EV, Makarov VV, Gintsburg AL (2019). Broad bactericidal activity of the Myoviridae bacteriophage lysins LysAm24, LysECD7, and LysSi3 against Gram-negative ESKAPE pathogens. Viruses.

[35] Antonova NP, Vasina DV, Rubalsky EO, Fursov MV, Savinova AS, Grigoriev IV (2020). Modulation of endolysin LysECD7 bactericidal activity by different peptide tag fusion. Biomolecules.

